# Surgical Aid Society

**Published:** 1894-12-15

**Authors:** 


### HOSPITAL MEETINGS.

### SURGICAL AID SOCIETY.

This society held its thirty-second annual meeting on Tuesday, December 11th, at the Cannon Street Hotel, the Lord Mayor in the chair.

The yearly report read by the secretary gave ample testimony to the excellent work done year by year on behalf of the poor and suffering, and in spite of the fact that the past year has been one of difficulty for nearly all charitable institutions, a continued growth in income is reported, and an increasingly large number of patients have benefited through the society's good offices. In order to meet the urgent demands for surgical appliances a certain portion of the invested funds, in respect of life subscribers, has been sold out, but the committee hope that the generosity of its friends will soon enable this amount to be replaced. Twelve thousand one hundred and fourteen patients have been assisted during the past year, and some 19,108 surgical appliances given away. A very specially helpful work is accomplished by means of the loan department, a large number of air and water beds, invalid carriages, &c., having been lent from the chief office, as well as from the local branches, to the immense comfort of many afflicted people. Alderman Samuel drew attention to the fact that the society was excluded from a share in the Hospital Sunday Fund, and asked if the Lord Mayor could do anything in this direction.

Mr. Bompas, Q.C., said that this society led to no encouragement of idleness; it tried to help those who helped themselves, and who were incapacitated by accident or illness, giving permanent relief from terrible suffering. He appealed to those who sacrificed money on personal luxuries to spend less on emulating their neighbours' expenditure and more on following the Christian duty of helping their poorer brethren.

Mr. William Grey, who has been treasurer of the society for many years, and is one of its warmest friends and supporters, was begged to continue his kind services, and the committee were duly re-elected.

Mr. Sheriff Hands commented on the fact that in spite of the establishment of new local branches, the expenses for stationery, salaries, &c., were less than last year, while the help given has increased, testifying to the good management of the society's funds. As no dinner is contemplated this year, the collection was made at the meeting, and a total sum of £353 was announced by the secretary. In conclusion the Lord Mayor said that so far as he could see there seemed no reason why the Surgical Aid Society should not receive a grant from the Hospital Sunday Fund, and he thought he might promise his support to their application. A grant was refused on a former occasion unless the committee would consent to abolish their system of subscribers' letters, and this they did not see their way to do. The system has, however, now been considerably modified, and, as a matter of fact, of the 19,108 appliances given away last year, 15,465 were supplied at once upon one subscriber's letter, and upon satisfactory evidence of a patient's inability to obtain more than a certain number of letters, his case is immediately recommended for help, so that the long delays which are the great objection to the letter system, are, in this case, practically non-existent.

The usual votes of thanks were warmly accorded to the medical officers of the society, and to the committee for their valuable services, and to the Lord Mayor for presiding at the meeting.

